# Activity Screening of Fatty Acid Mimetic Drugs Identified Nuclear Receptor Agonists

**DOI:** 10.3390/ijms231710070

**Published:** 2022-09-03

**Authors:** Moritz Helmstädter, Simone Schierle, Laura Isigkeit, Ewgenij Proschak, Julian Aurelio Marschner, Daniel Merk

**Affiliations:** 1 Institute of Pharmaceutical Chemistry, Goethe University Frankfurt, 60438 Frankfurt, Germany; 2 Department of Pharmacy, Ludwig-Maximilians-Universität München, 81377 Munich, Germany

**Keywords:** transcription factor, polypharmacology, selective optimization of side activities, oxaprozin, bortezomib, tianeptine, mycophenolate

## Abstract

Fatty acid mimetics (FAM) are bioactive molecules acting through the binding sites of endogenous fatty acid metabolites on enzymes, transporters, and receptors. Due to the special characteristics of these binding sites, FAMs share common chemical features. Pharmacological modulation of fatty acid signaling has therapeutic potential in multiple pathologies, and several FAMs have been developed as drugs. We aimed to elucidate the promiscuity of FAM drugs on lipid-activated transcription factors and tested 64 approved compounds for activation of RAR, PPARs, VDR, LXR, FXR, and RXR. The activity screening revealed nuclear receptor agonism of several FAM drugs and considerable promiscuity of NSAIDs, while other compound classes evolved as selective. These screening results were not anticipated by three well-established target prediction tools, suggesting that FAMs are underrepresented in bioactivity data for model development. The screening dataset may therefore valuably contribute to such tools. Oxaprozin (RXR), tianeptine (PPARδ), mycophenolic acid (RAR), and bortezomib (RAR) exhibited selective agonism on one nuclear receptor and emerged as attractive leads for the selective optimization of side activities. Additionally, their nuclear receptor agonism may contribute relevant and valuable polypharmacology.

## 1. Introduction

Fatty acids (FA) are an important source of energy and are key components of lipid bilayers, thus contributing essentially to physiological homeostasis [1]. In addition, several FAs and FA metabolites act as signaling molecules on various types of receptors [1,2,3,4]. These receptors, as well as the enzymes metabolizing FAs to FA-derived signaling molecules, are attractive macromolecular targets in multiple indications, and many are already addressed by approved drugs [2]. Complementary to the structural features of FAs (and their metabolites), FA binding receptors and enzymes comprise binding sites with certain commonalities. FA binding sites typically are highly hydrophobic apart from a site for a strong ionic contact of the acid function as an often exclusive directed interaction [2]. Drugs targeting FA binding sites have to adapt to these natural prerequisites and thus comprise common features, too. They resemble FAs in terms of structural architecture with a bulky hydrophobic skeleton carrying a polar acidic head group [2]. Bioactive compounds of this structural composition that target binding sites of natural FAs and their metabolites are FA mimetics (FAM) [2].

The binding of FAs and FAMs to their macromolecular binding sites, apart from the common ionic contact, is mainly driven by hydrophobic interactions, since opportunities for polar contacts are rare [2]. Selectivity among FA(M) binding sites, therefore, mainly depends on shape and complementarity with the (intended) interaction site. The lack of directed contacts defining the ligand–target interaction in FA binding proteins together with the conformational dynamics of these proteins may suggest that the selectivity of synthetic FAMs may be limited. They may thus exhibit unanticipated biological activities, the discovery of which would potentially uncover valuable polypharmacological profiles [5]. Moreover, off-target interactions of FAMs may open opportunities for the selective optimization of side activities (SOSA) [6,7], which refers to the development of a new drug from an old drug by inverting the main-target/off-target potencies. This concept has the advantage that the lead (the original “old” drug) is known to have favorable features and to be safe, thus potentially speeding up drug discovery [6,7]. Aiming to reveal unknown polypharmacology among FAM drugs on FA-activated transcription factors and to discover leads for SOSA, we have screened a custom collection of marketed and late-stage experimental drugs with an FAM structure in vitro. We have focused on the modulation of the retinoic acid receptor (RAR), peroxisome proliferator-activated receptors (PPARs), the farnesoid X receptor (FXR), and the retinoid X receptor (RXR) which are endogenously activated by FA metabolites and related lipids with acidic groups. Additionally, the nuclear liver X receptor (LXR) and the vitamin D receptor (VDR) binding lipids, rather than FAs, were included for comparison and to reveal potential general lipophilicity-driven promiscuity. We have discovered unanticipated structural features associated with selectivity and promiscuity. Moreover, the screening has revealed remarkable off-target activities of a number of approved drugs, rendering them as attractive leads for SOSA.

## 2. Results

**Figure 1:**
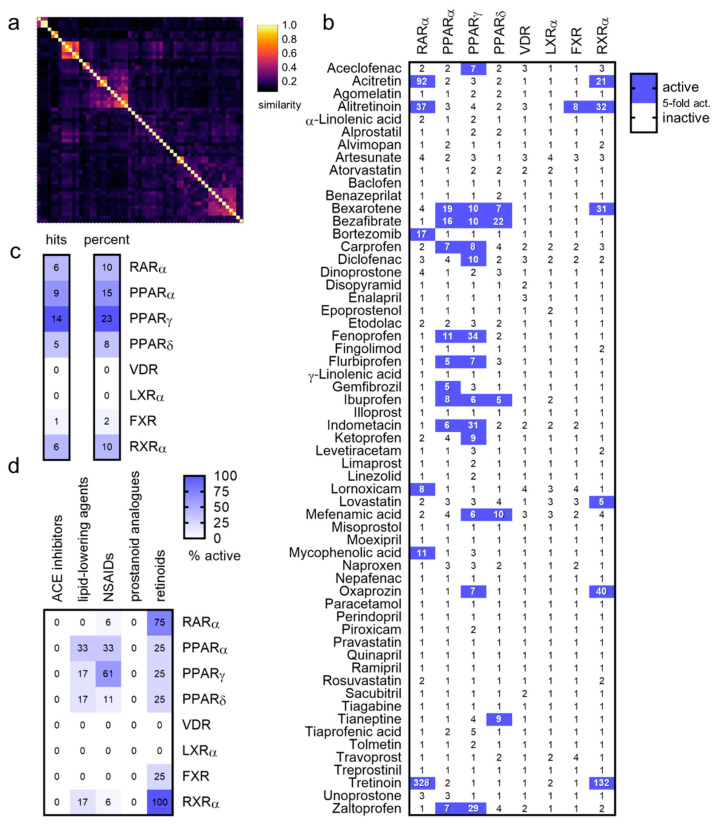
Fatty acid mimetic (FAM) drug library screening. (**a**) Despite their common fatty acid mimetic architecture, the screened FAM drugs displayed high chemical diversity. The heatmap shows the pairwise Tanimoto similarity computed on Morgan fingerprints [17]. (**b**) Results of the primary screening of FAM drugs on eight nuclear receptors. Heatmap shows the mean fold nuclear receptor activation, *n* = 2. Compounds causing ≥ 5-fold activation were considered active. (**c**) The screening results indicated different promiscuity of nuclear receptors in terms of tolerated FAM ligands. PPARγ was activated by the greatest number of FAMs, while only a few hits were retrieved for PPARδ and FXR. (**d**) The screening revealed promiscuity of NSAIDs and retinoids among nuclear receptors, while ACE inhibitors and prostanoid analogues were highly selective.

FAMs for screening on FA- and lipid-activated nuclear receptors were selected from the DrugBank [8] based on a FAM structure [2] composed of a lipophilic scaffold with a carboxylic acid or an acidic bioisostere. Based on these criteria, a total of 64 drugs (full list in Table S1) were selected. FAM drugs (montelukast, pranlukast, zafirlukast, and lesinurad) which have been systematically studied for nuclear receptor agonism previously [9,10,11] were not included. Descriptor analysis compared to the ChEMBL [12] library demonstrated compliance of the screening set with typical features of FAMs [2] (Figure S1). Nevertheless, despite sharing common descriptors and the presence of closely related compound clusters (retinoids, phenylpropanoic acid based non-steroidal anti-inflammatory drugs (NSAIDs), and prostanoid analogues), the collection of FAM drugs exhibited high chemical diversity (Figure 1a).

The FAM collection was tested for the activation of RAR, PPAR, VDR, LXR, FXR, and RXR in hybrid reporter gene assays [13], providing a uniform in vitro test system for all studied receptors. These assays employ a chimeric receptor protein to control the reporter gene expression, which is composed of the ligand binding domain (LBD) of the respective human nuclear receptor and the DNA binding domain of the yeast protein Gal4. A firefly luciferase reporter construct comprising five tandem repeats of the Gal4 response element was used as the reporter, and constitutively expressed renilla luciferase (SV40 promoter), served for normalization and toxicity control. The assays were conducted in transiently transfected HEK293T cells in 96-well format. The 64 drugs with FAM structures (Table S1, 50 µM each) were tested in this setting in two independent experiments in duplicates. Five compounds exhibited toxic or non-specific promiscuous effects and were excluded.

Generally, the screened FAMs revealed few activities on nuclear receptors even at the high 50 µM test concentration (Figure 1b). Despite covering only a small subset of FA binding proteins, the screening thus indicated a rather high selectivity of FAM drugs. Among the 59 remaining non-toxic FAMs, 22 activated at least 1 of the 8 studied nuclear receptors (≥5-fold activation). Half of the active hits (11 compounds) acted as agonists on only one of the studied receptors, while the other half exhibited more promiscuous profiles and activated up to four receptors.

Among the eight studied receptors, PPARγ was activated by the greatest number of FAMs, followed by its close relative PPARα (Figure 1c). Of note, the screening collection contained no drugs specifically approved as PPARγ agonists (two for PPARα: bezafibrate and gemfibrozil). These results therefore align with high promiscuity of PPARγ (comprising the largest binding pocket of the PPARs [14]) regarding the types of accepted ligands for which it has been included in safety and promiscuity panels [15,16].

Analysis of the screening results by drug classes (Figure 1d) revealed interesting differences in terms of promiscuity towards nuclear receptors. Five drug classes were represented in the screening set with more than two examples: angiotensin converting enzyme (ACE) inhibitors (6), lipid-lowering agents (6), NSAIDs (18), prostanoid analogues (9), and retinoids (4). ACE inhibitors and prostanoid analogues turned out to be highly selective over nuclear receptors. None of these drugs activated the studied receptors. Some activities of lipid-lowering agents on nuclear receptors were observed, which corresponded to known PPAR agonism of fibrates, however, and retinoids activated RAR and RXR, which reflected their known mode of action, too. NSAIDs emerged as the most promiscuous drug class and revealed multiple activities on PPARs. More than 60% (11 out of 18) of the studied NSAIDs exhibited PPARγ agonism, and 33% (6) also activated PPARα. PPARδ agonism with 11% of NSAIDs (2) was less prevalent. The NSAID oxaprozin deviated from the trend of promiscuous PPAR agonism but robustly activated RXR.

**Figure 2:**
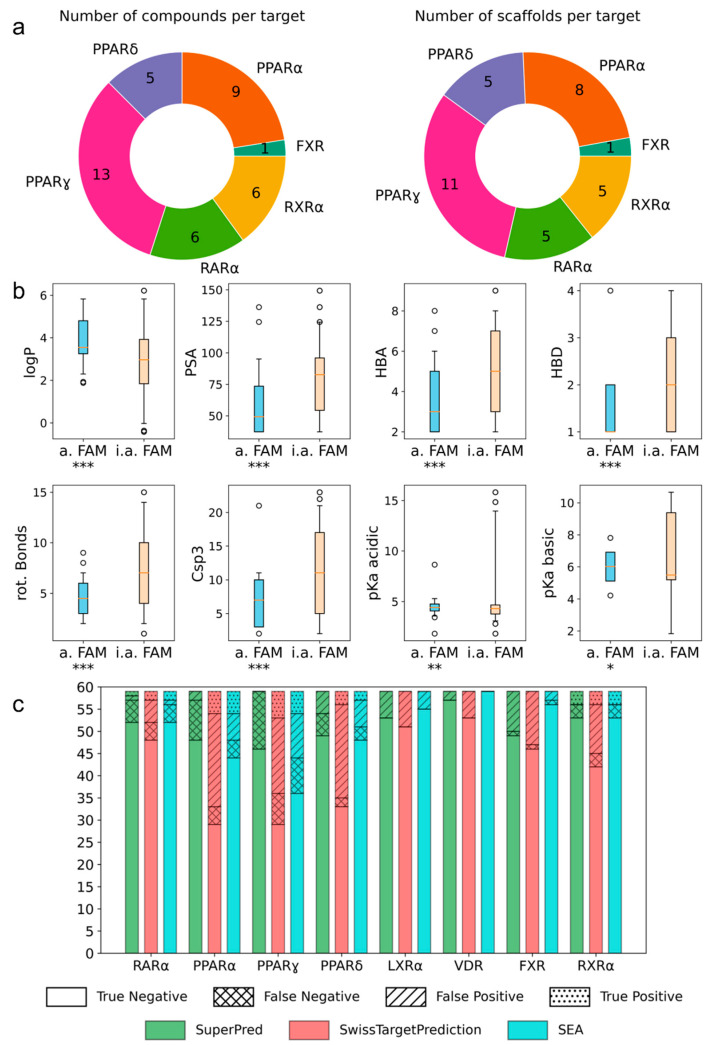
Molecular features of the screening hits. (**a**) The activity screening yielded structurally novel and chemically diverse hits for six nuclear receptors. (**b**) FAM drugs active on nuclear receptors had a higher logP, a lower PSA, less HBA and HBD, less rotatable bonds, and a lower Csp3 fraction than the inactive compounds. * *p* < 0.05, ** *p* < 0.01, *** *p* < 0.001 (t-test). (**c**) The target prediction tools SuperPred [19], SwissTargetPrediction [20], and SEA [21] did not reproduce the screening results, suggesting that the dataset is a valuable addition for model training.

The screening interestingly revealed pronounced differences in the promiscuity of the FAMs. To extract features driving selectivity or promiscuity among nuclear receptors, we compared the characteristics of active hits with inactive FAMs in the collection (Figure 2). Due to the high structural diversity of the FAM screening set (Figure 1a), almost all actives represented an individual scaffold (Figure 2a), thus preventing a correlation between activities and scaffolds. Analysis of molecular features (Figure 2b) revealed higher lipophilicity (logP), lower polar surface area, and less H-bond donors and acceptors for FAMs activating the NR (≥5-fold activation) compared to the inactive FAMs. Moreover, a lower number of rotatable bonds and less sp3 carbon atoms favored activity on the NR, thus suggesting that more rigid FAMs exhibit activity on more targets. As the binding of FAMs to their targets is often driven by the hydrophobic effect, which comes along with a loss of degrees of freedom, more rigid FAMs have a thermodynamic advantage in binding [2,18], which also aligns with a more rigid FAM binding to more targets.

As the screening revealed interesting activities of several drugs on new macromolecular targets, we probed whether well-established target prediction tools (SuperPred [19], SwissTargetPrediction [20], and SEA [21]) would have suggested these activities. Interestingly, high rates of false-negative and false-positive predictions were retrieved from all three tools (Figure 2c). Precision, referring to the rate of true actives among predicted actives, and sensitivity, referring to the fraction of correctly predicted actives among all actives, of the prediction tools were low for the FAM screening set (SuperPred: sensitivity 0.10 and precision 0.14; SwissTargetPrediction: sensitivity 0.48 and precision 0.16; SEA: sensitivity 0.43 and precision 0.37). This overall performance suggested the hits from our screening being outside the chemical space covered by these models. These results highlight that the FAM activity dataset is a valuable addition to model training for target prediction on nuclear receptors (full dataset is available as a csv in the Supporting Information).

Four FAMs emerged from the screening by exhibiting selective agonism on one of the studied nuclear receptors. Bortezomib and mycophenolic acid selectively activated RAR, oxaprozin acted as an RXR agonist (and a weak PPARγ agonist), and tianeptine exhibited PPARδ agonism. To estimate the pharmacological relevance of these activities and probe the potential of these compounds for SOSA, we recorded full dose-response curves on the respective targets (Table 1). Oxaprozin was confirmed as an RXR agonist [22] with intermediate potency, and tianeptine exhibited PPARδ agonism with considerable activation efficacy. Bortezomib and mycophenolic acid activated RAR with low micromolar EC_50_ values despite weak activation efficacy. All four drugs, therefore, are attractive leads for SOSA. For oxaprozin, this concept has already been implemented successfully leading to the development of highly potent and selective RXR agonists [22].

Structurally, the RAR agonism of bortezomib and PPARδ activation by tianeptine are particularly interesting due to their untypical molecular features with respect to FAMs. The boronic acid motif contained in bortezomib is still uncommon among approved drugs but is continuously gaining attention in medicinal chemistry for its special characteristics [23,24]. For example, boronic acids possess unique H-bonding potential, can reversibly form covalent adducts with various nucleophiles, and convert between trigonal, planar, and tetrahedral geometries [23,24]. The RAR agonism of bortezomib with considerable potency and selectivity therefore suggests the boronic acid group as potential motif to be considered in future FAM and nuclear receptor ligand development. Additionally, the PPARδ agonism of tianeptine is remarkable since PPAR agonists typically comprise highly lipophilic backbones, while tianeptine is favorably polar (logP 1.54) and contains a basic amine and a polar sulfonamide in its lipophilic tail. It may therefore enable the design of new PPARδ agonists with preferable physicochemical features. To obtain molecular insights into RAR modulation by bortezomib and mycophenolic acid as well as the PPARδ agonism of tianeptine, we evaluated their interactions by molecular docking. Oxaprozin was omitted as a crystal structure of an analogue with RXRα and has been solved (pdb ID: 7b9o) [22].

**Figure 3:**
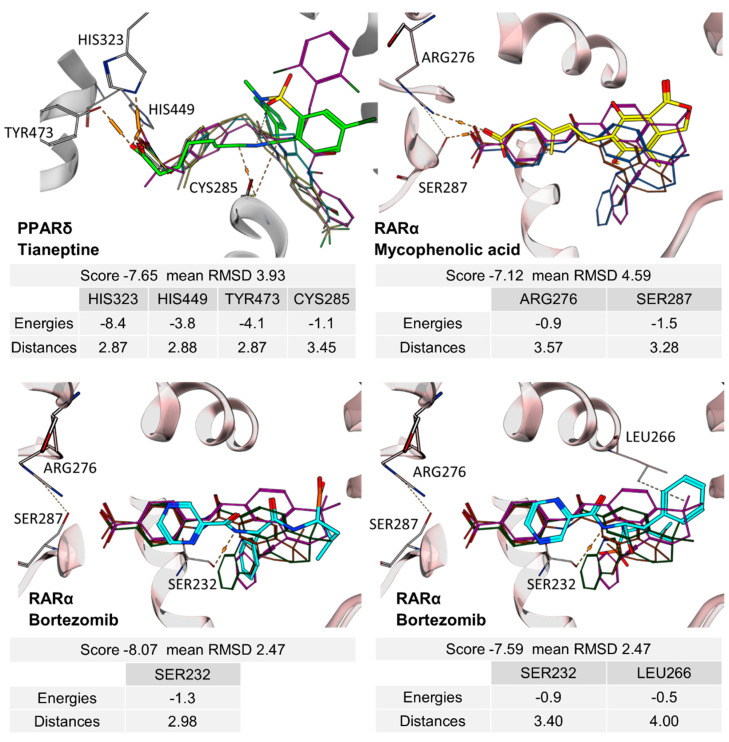
Predicted interactions of tianeptine (green), mycophenolic acid (yellow), and bortezomib (cyan) with their respective nuclear receptor targets. Docking was performed in MOE using X-ray structures of the PPARδ LBD (pdb ID: 1GWX [25]) and the RARα LBD (pdb ID: 3KMZ) [26] as templates. Co-crystalized reference ligands (PPARδ: magenta—GW2433; cyan—L165041; rose—GW501516; and yellow—GW0742; RARα: magenta—BMS493; brown—Ro 40-6055; and green—BMS614) are shown for comparison.

The docking (Figure 3) of Tianeptine to the PPARδ LBD (pdb ID: 1GWX [25]) suggested a typical PPAR agonist binding mode with strong contacts of the carboxylic acid motif to the activation function represented by His323, His449, and Tyr473 and with the dihydrodibenzo[c,f][1,2]thiazepine system favorably bound in the spacy lipophilic pocket of PPARδ. For the interaction of mycophenolic acid with RARα, molecular docking also suggested polar interactions of the carboxylic acid with Arg276 and Ser287, which is common among RAR agonists. Apart from these directed contacts, mycophenolic acid binding appeared to be mainly mediated by hydrophobic interactions with the highly lipophilic RAR ligand binding site, as expected for a FAM. The docking of bortezomib to the RARα ligand binding site interestingly revealed two reasonable binding modes, neither of which indicated an interaction of the boronic acid motif with Arg276 and Ser287. Apart from an H-bond from the pyrazinecarboxamide’s nitrogen atom to the Ser232 side chain observed in both poses, the binding of bortezomib was mediated by hydrophobic contacts. Compared to the respective co-crystalized ligands, tianeptine, mycophenolic acid, and bortezomib revealed lower docking scores by several orders of magnitude, which is in-line with their intermediate potencies on PPARδ and RAR. Thus, the predicted binding modes suggest considerable potential to optimize affinity to the nuclear receptor targets further supporting the suitability of the drugs for SOSA.

**Figure 4:**
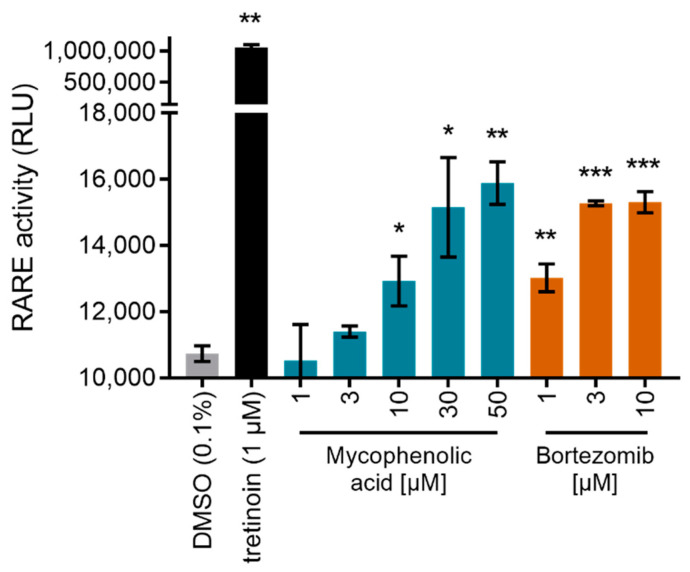
Mycophenolic acid and bortezomib activated transcriptional activity via the human RAR response element (RARE). Data are the mean ± S.E.M., *n* = 3. * *p* < 0.05, ** *p* < 0.01, *** *p* < 0.001 (*t*-test vs. DMSO).

While the moderate PPARδ agonism of tianeptine likely has no pharmacological relevance at the recommended low dose (12.5 mg), RAR activation by bortezomib and mycophenolic acid may contribute to their pharmacological activity. The proteasome inhibitor, bortezomib, is approved for multiple myeloma treatment [27]. RAR agonists have been intensively studied in the same indication with promising results [28,29,30], suggesting a beneficial dual mode-of-action for bortezomib. Interestingly, sensitization of myeloma cells to proteasome inhibitors by concomitant retinoic acid treatment has recently been reported [31]. Similarly, RAR activation by the immunosuppressant inosine monophosphate dehydrogenase (IMPDH) inhibitor, mycophenolic acid [32], is remarkable since RARs (together with RXRs) are critical regulators of immunity [33]. Importantly, clinical pharmacokinetics studies in healthy volunteers have reported peak plasma concentrations of mycophenolic acid between 24.5 mg/L and 32.8 mg/L after oral administration [34] corresponding to 77 to 103 µM. Bortezomib reached maximum plasma concentrations of 112 mg/L [35] corresponding to 29 µM. Hence, the reported plasma levels of both drugs markedly exceed their EC_50_ values for RAR activation. To characterize the RAR agonism of bortezomib and mycophenolic acid further in a more physiological setting, we tested their ability to induce transcription via the human RAR response element (RARE). Both compounds indeed activated RARE at low micromolar concentrations, supporting potential pharmacological relevance despite low efficacy compared to the full RAR agonist tretinoin (Figure 4).

## 3. Discussion

Due to the special requirements for mimicking FAs and binding to their binding sites, FAMs share common chemical characteristics and represent an important class of bioactive molecules. As FAs exhibit critical roles as signaling molecules, FAMs can be employed for pharmacological interference with multiple regulatory systems and thus have an importance as drugs. The collection of proteins endogenously modulated by FAs includes several nuclear receptors, which act as ligand-activated transcription factors. These proteins regulate gene expression and their activation can have a major pharmacological impact. Accordingly, nuclear receptor modulation is a well-established therapeutic strategy and mediates the effects of essential medicines, for example, glucocorticoids. The FA-binding nuclear receptors covered by our screening are particularly involved in the regulation of nutrient homeostasis, inflammation, and proliferation. While several potent drugs such as rosiglitazone and bexarotene have been developed to pharmacologically activate these receptors, the potential promiscuity of other approved FAM drugs on FA activated transcription factors remained elusive. The knowledge of the side activities of FAMs on nuclear receptors could, however, improve the understanding of their pharmacological profiles and open avenues for SOSA.

To obtain systematic insights into the promiscuity or selectivity of FAM drugs among FA-binding nuclear receptors, we have conducted an activity screening in a uniform assay panel. In addition to revealing several leads for SOSA, this screening has shown considerable promiscuity of NSAIDs on PPARγ, while several other types of FAMs were highly selective over nuclear receptors. Interestingly, the results of the activity screening were not anticipated by three well-established target prediction tools, potentially suggesting that the chemical space of FAMs is insufficiently represented in compound/bioactivity data, which are used for model development. Our screening data may thus provide a valuable addition to publicly available training data. For the RAR agonism of mycophenolic acid and bortezomib, the screening revealed new pharmacodynamic effects possibly contributing to the pharmacological profiles of these drugs, which further evaluation will have to confirm.

Overall, while highlighting the majority of this compound class as selective, screening FAM drugs for nuclear receptor activation revealed several unprecedented activities that may valuably contribute to polypharmacological profiles or offer attractive starting points for SOSA, as exemplified by the development of RXR agonists from oxaprozin.

## 4. Materials and Methods

**Screening compounds**. The FAM drugs were obtained from commercial vendors.

**Hybrid reporter gene assays**. Hybrid reporter gene assays were performed in transiently transfected HEK293T cells (German Collection of Microorganisms and Cell Culture GmbH, DSMZ), as described previously [36], using the hybrid receptor plasmids pFA-CMV-hRARα-LBD [37], pFA-CMV-hPPARα-LBD [38], pFA-CMV-hPPARγ-LBD [38], pFA-CMV-hPPARδ-LBD [38], pFA-CMV-hVDR-LBD [39], pFA-CMV-hLXRα-LBD [40], pFA-CMV-hFXR-LBD [41], and pFA-CMV-hRXRα-LBD [42]. pFR-Luc (Stratagene, La Jolla, CA, USA) served as reporter, and pRL-SV40 (Promega, Madison, WI, USA) was used as an internal control. Transient transfection was performed with Lipofectamine LTX reagent (Invitrogen, Carlsbad, CA, USA); luciferase activity was measured using the Dual-Glo Luciferase Assay System (Promega) according to the manufacturer’s protocol and a Tecan Spark luminometer (Tecan Group AG, Männedorf, Switzerland). Firefly luminescence was divided by Renilla luminescence and multiplied by 1000, resulting in relative light units (RLU) to normalize for transfection efficiency and cell growth. Fold activation was obtained by dividing the mean RLU of the test compound by the mean RLU of the untreated control. For the initial drug fragment screening, the FAM library was tested in two biologically independent experiments in duplicates. Follow-up experiments were conducted in at least three biologically independent experiments in duplicates. For dose–response curve fitting and calculation of EC_50_ values, the equation “[Agonist] versus response (variable slope-four parameters)” was performed in GraphPad Prism (version 7.00, GraphPad Software, La Jolla, CA, USA) with the mean fold activation values ± S.E.M. Tretinoin, GW7647, pioglitazone, L165041, calcitriol, TO901317, GW4064, and bexarotene (each at 1 µM) were used as reference agonists.

**Reporter gene assay for RARE activation**. Activation of the human RAR response element was studied in transiently transfected HEK293T cells using the reporter plasmid pGL3-RARE-luciferase [43] (Addgene plasmid #13458, Watertown, MA, USA), the receptor plasmids pcDNA3.1-hRARα [44] (Addgene plasmid #135397), and pSG5-hRXR [45] and pRL-SV40 (Promega) as internal control. Transient transfection was performed with Lipofectamine LTX reagent (Invitrogen); luciferase activity was measured using the Dual-Glo Luciferase Assay System (Promega) according to the manufacturer’s protocol and a Tecan Spark luminometer (Tecan). Firefly luminescence was divided by Renilla luminescence and multiplied by 1000, resulting in relative light units (RLU) to normalize for transfection efficiency and cell growth. Experiments were conducted in three biologically independent experiments in duplicates. Tretinoin (1 µM) was used as the reference RAR agonist.

**Molecular feature analysis and target prediction**. Pairwise Tanimoto similarities and Morgan fingerprints were calculated using RDKit version 2022.03.2 (RDKit: Open-source cheminformatics; http://www.rdkit.org/ accessed on 21 August 2022) in python v3.7 (Python Software Foundation, https://www.python.org/ accessed on 21 August 2022); molecular features logP, PSA, HBA, HBD, rotatable bonds, and Csp3 were calculated with DataWarrior version 5.2.1 [46] The values for pKa acidic/basic were retrieved from DrugBank version 5.1.9 [8] and ChEMBL [12]. The statistical significance between sets of active and inactive molecules was calculated using python v3.7 and the module scipy.stats v1.7.3 [47]. Target predictions were performed with the prediction tools SuperPred [19], SwissTargetPrediction [20], and SEA [21], and the results were analyzed in KNIME 4.6.1 (KNIME AG, Zurich, Switzerland). Results were visualized in python v3.7 using the module matplotlib v3.5.1 [48].

**Molecular docking**. Molecular docking was performed in Molecular Operating Environment (MOE, version 2022.02, Chemical Computing Group Inc. Montreal, QC, Canada). The X-ray structure of the PPARδ LBD in complex with GW2433 (pdb ID: 1GWX) [25] and the RARα LBD in complex with BMS493 (pdb ID: 3KMZ) [26] served as templates. The structures were prepared using the MOE QuickPrep tool with default settings, adjusting the protonation states of the complexes. Compounds were prepared using the MOE Wash tool with the dominant protonation state at pH 7.0; coordinates were rebuilt in 3D, and existing chirality was maintained. The following settings were used for all docking calculations: Force Field = Amber10:EHT; Receptor = Receptor and Solvent Atoms; Site = Ligand Atoms of crystallized ligand; Placement = Triangle Matcher; Refinement = Induced Fit; first scoring function = London dG with 100 poses; and second scoring function = GBVI/WSA dG with 10 poses for each compound. Redocking of the crystallized ligand GW2433 in PPARδ (RMSD = 1.86, mean RMSD = 3.30, and score = −11.88) and BMS493 in RARα (RMSD = 0.16, mean RMSD = 1.50, and score = −11.00) confirmed suitability of the method.

## Figures and Tables

**Table 1:** In vitro activity of FAMs with possibly relevant side-target activity and with potential for SOSA ^1^.

Drug (Structure, INN)	Activity
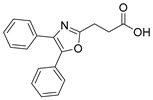 Oxaprozin	partial RXR agonist [22] EC_50_ = 16.1 ± 0.6 µM (23 ± 1% max. act.) (RXRα)
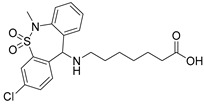 Tianeptine	partial PPARδ agonist EC_50_ = 28 ± 4 µM (43 ± 4% max. act.)
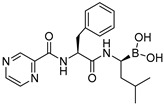 Bortezomib	partial RAR agonist EC_50_ = 0.8 ± 0.3 µM (<10% max. act.) (RARα)
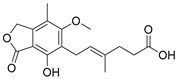 Mycophenolic acid	partial RAR agonist EC_50_ = 1.5 ± 0.6 µM (<10% max. act.) (RARα)

^1^ Activities were determined in Gal4 hybrid reporter gene assays. Maximum relative activation refers to the activity of the reference agonists bexarotene (RXR), L165041 (PPARδ), and tretinoin (RAR), each at 1 µM. Data are the mean ± S.E.M., *n* = 3.

## Data Availability

The in vitro data associated with this study are available as .csv file in the Supporting Information.

## Supplementary Materials

The supporting information can be downloaded at: https://www.mdpi.com/article/10.3390/ijms231710070/s1.
