# Supplementary figures and images for: Activity Screening of Fatty Acid Mimetic Drugs Identified Nuclear Receptor Agonists
Source: Int J Mol Sci. 2022 Sep 3;23(17):10070. doi: 10.3390/ijms231710070 (PMC9456086; doi:10.3390/ijms231710070)

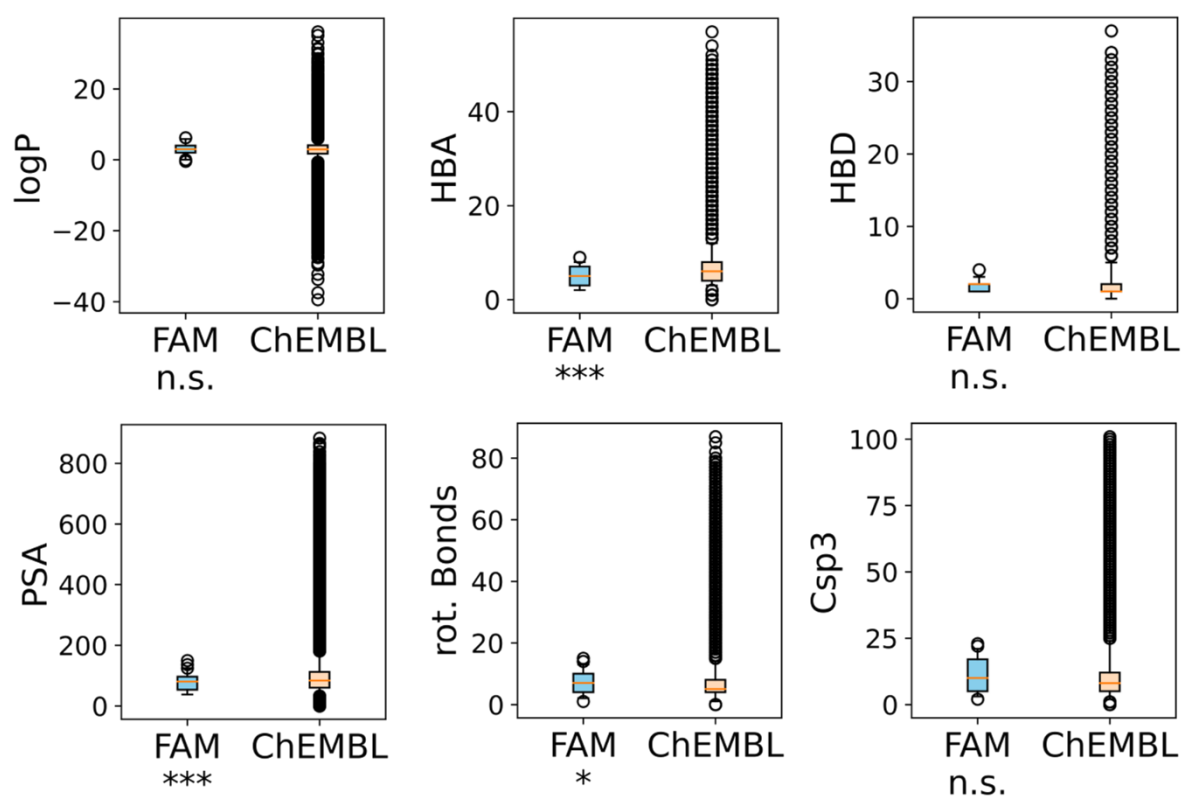

**Figure S1.** Basic molecular descriptors of FAM drugs compared to ChEMBL compounds. \*  $p < 0.05$ , \*\*\*  $p < 0.001$  (ttest).

Supplement: Supplementary file 1 [file ijms-23-10070-s001.zip › Figure S1.pdf]
